# Association between quetiapine use and self-harm outcomes among people with recorded personality disorder in UK primary care: A self-controlled case series analysis

**DOI:** 10.1177/02698811221131990

**Published:** 2022-11-01

**Authors:** Joseph F Hayes, Sarah Hardoon, Jessica Deighton, Essi Viding, David PJ Osborn

**Affiliations:** 1 Division of Psychiatry, University College London, London UK; 2 Camden and Islington NHS Foundation Trust, London, UK; 3 Anna Freud Centre, London, UK; 4 Division of Psychology and Language Sciences, University College London, London, UK

**Keywords:** Personality disorder, quetiapine, self-harm

## Abstract

**Background:**

Quetiapine is frequently prescribed to people with personality disorder diagnoses, but this is not supported by evidence or treatment guidelines.

**Aims:**

To examine associations between periods of quetiapine prescribing and self-harm events in people with personality disorder.

**Method:**

Self-controlled case series using linked primary care and hospital records covering the period 2007–2017. We calculated incidence rates and incidence rate ratios (IRRs) for self-harm events during periods when people were prescribed (exposed to) quetiapine, as well as periods when they were unexposed or pre-exposed to quetiapine.

**Results:**

We analysed data from 1,082 individuals with established personality disorder diagnoses, all of whom had at least one period of quetiapine prescribing and at least one self-harm episode. Their baseline rate of self-harm (greater than 12 months before quetiapine treatment) was 0.52 episodes per year. Self-harm rates were elevated compared to the baseline rate in the month after quetiapine treatment was commenced (IRR 1.85; 95% confidence interval (CI) 1.46–2.34) and remained raised throughout the year after quetiapine treatment was started. However, self-harm rates were highest in the month prior to quetiapine initiation (IRR 3.59; 95% CI 2.83–4.55) and were elevated from 4 months before quetiapine initiation, compared to baseline.

**Conclusion:**

Self-harm rates were elevated throughout the first year of quetiapine prescribing, compared to the baseline rate. However, rates of self-harm reduced in the month after patients commenced quetiapine, compared to the month before quetiapine was initiated. Self-harm rates gradually dropped over a year of quetiapine treatment. Quetiapine may acutely reduce self-harm. Longer-term use and any potential benefits need to be balanced with the risk of adverse events.

## Introduction

Approximately 12% of the general population are estimated to meet criteria for a personality disorder, a pattern of personality traits that are maladaptive and inflexible, and result in significant distress and functional impairment (Widiger, 2012). A number of studies have highlighted the shortcomings of categorical distinctions between personality disorder subtypes (Widiger and Trull, 2007) and these distinctions have been removed in the 11th version of the International Classification of Diseases (ICD-11) (Oltmanns and Widiger, 2019).

Pharmacological approaches to managing symptoms associated with personality disorder are commonly delivered in clinical practice. However, these treatments are not supported by high-quality evidence regarding their effectiveness and they are not recommended routinely in the majority of treatment guidelines for personality disorder (Hancock-Johnson et al., 2017; Stoffers and Lieb, 2015; Stoffers-Winterling et al., 2020). The United Kingdom (UK) National Institute for Health and Care Excellence guidelines state that antipsychotic medication should not be used for the medium- to long-term treatment of antisocial or borderline personality disorder (National Institute for Health and Care Excellence 2009a, 2009b). We recently found that 25% of people in UK primary care with a personality disorder diagnosis (and no diagnostic code suggesting psychotic illness) have been prescribed antipsychotic medication (Hardoon et al., 2022). Quetiapine (a dopamine and serotonin receptor antagonist, and norepinephrine transporter inhibitor) is the antipsychotic medication most commonly prescribed in this group.

There has been one randomised controlled trial of quetiapine for borderline personality disorder for which results are available (Black et al., 2014; Lee et al., 2016). This trial of 95 participants found that daily doses of 150 and 300 mg were associated with reduced psychological distress, interpersonal sensitivity, depression and hostility at 8 weeks compared to placebo. There was no clear difference between doses, but there were higher rates of adverse events in the group receiving 300 mg per day. There have also been a number of small open-label studies for the treatment of borderline personality disorder, that suggest some effect on impulsivity and affective symptoms (Bellino et al., 2006; Gruettert and Friege, 2005; Perrella et al., 2007; Romine et al., 2008; Van den Eynde et al., 2008; Villeneuve and Lemelin, 2005).

Self-harm is common in patients receiving a diagnosis of personality disorder (Krysinska et al., 2006), particularly borderline or emotionally unstable personality disorder. There are case series’ reporting improvements in self-harming behaviour in adolescents treated with quetiapine (Good, 2006; Pathak et al., 2005). However, observational studies in patients with bipolar disorder and schizophrenia have not found quetiapine prescribing to be associated with reduced self-harm (Jefsen et al., 2021; Ma et al., 2018). We are aware of no studies that examine quetiapine’s effects on self-harm in people with personality disorder diagnoses. However, it is postulated that quetiapine may alleviate symptoms of self-harm in people with personality disorder via effects on impulsivity and aggression (Smith, 2005). Trials of quetiapine as an anti-suicidal agent in personality disorder are likely to be prohibitive due to issues of power, follow-up and risk, whereas observational designs such as the self-controlled case series (SCCS) can potentially address this pressing clinical issue with large, representative samples while minimising confounding.

We investigated the association between quetiapine prescribing and self-harm events among people with a recorded diagnosis of personality disorder using linked UK primary care and hospital records. We hypothesised that to be clinically useful, quetiapine treatment would be associated with reduced rates of self-harm in individuals with personality disorder compared to their baseline rates of self-harm when unexposed.

## Methods

### Study design

We used the SCCS study design (Petersen et al., 2016). The principle of the SCCS method is that when assessing the impact of an exposure against being unexposed, individuals are their own controls. This has the key advantage that all fixed observed and unobserved confounders (e.g. sociodemographic characteristics) will be automatically accounted for in the analysis. Time-varying confounders such as age can be adjusted for in the SCCS analysis. In an SCCS analysis only individuals from the study population who (i) experience the outcome and (ii) have both exposed and unexposed time periods during follow-up are included (Whitaker et al., 2006). Hence our SCCS design included people with a recorded diagnosis of personality disorder and at least one record of our outcome, self-harm. They also needed to have a period of quetiapine treatment to allow us to compare their self-harm rates in different periods when they were prescribed, and not prescribed, quetiapine.

### Data source

We used data from the Clinical Practice Research Datalink (CPRD), which comprises computerised, anonymised longitudinal patient records retrieved from participating general practices across the UK. At the time of analysis patient records up to 2018 were available. The CPRD includes two separate databases: CPRD Gold and CPRD Aurum (Herrett et al., 2015; Wolf et al., 2019). General practices contribute to Gold or Aurum depending on the computer software package used by the practice for the computerised patient records: practices using Vision software are included in Gold, while practices using EMIS software are included in Aurum. The content of the two databases are similar; including all diagnoses, symptoms and other health data (blood tests, health indicators) recorded by the general practitioner in computerised records, as well as all medication prescriptions.

The CPRD primary care records were also linked by the data providers to secondary care records from Hospital Episode Statistics (HES) data, including records from accident and emergency (A&E) (records available from 1 April 2007 to 31 December 2017), admitted patient care (APC) (1 April 1997 to 31 December 2017) and operations (1 April 2003 to 31 December 2017) (Herbert et al., 2017; Padmanabhan et al., 2019). Linkage is limited to consenting general practices in England.

### Study population: People with a record of personality disorder and an episode of self-harm

We included people who had a record in their general practice notes of a diagnosis of personality disorder after the age of 18. A list of relevant codes was developed using established search techniques involving searching for relevant terms, and with reference to version 10 of the International Classification of Diseases (ICD-10) sections F60–F61 (Davé and Petersen, 2009). The complete code list is available on request.

All personality disorder codes were categorised in line with ICD-10 as: paranoid, schizoid, dissocial, emotionally unstable (including borderline), histrionic, anankastic, anxious/avoidant, dependent and other (chiefly comprising codes for non-specific personality disorder, but also including rare diagnoses such as eccentric, haltlose, immature, narcissistic, masochistic, passive-aggressive and mixed). Where an individual had more than one type of specific personality disorder record, the most recent record was assigned as their diagnosis.

We excluded individuals with a record of severe mental illness, including schizophrenia, bipolar disorder and other non-organic psychotic illness before the study start date. Individuals who received an severe mental illness (SMI) diagnosis during follow-up, had follow-up time censored at the date of SMI diagnosis.

### Follow-up period

Start of follow-up was the latest of 1 April 2007, date of registration at CPRD practice or date of first personality disorder record, *plus* 3 months to allow for definition of exposure (quetiapine) status at baseline. End of follow-up was defined as the earliest of 31 December 2017, date of death, date of transfer out of CPRD practice, last date of the practice contributed data to CPRD, first SMI record or latest date or which HES linkage data were available. The dates 1 April 2007 and 31 December 2017 reflect the period over which linked data from HES overlaps with data from CPRD.

### Outcome – Episodes of self-harm

Our outcome was a record of a self-harm event (including all self-harm events and suicide attempts and completed suicide) either in primary care or HES. Individuals could have multiple self-harm events. In CPRD, we used codes corresponding to self-harm to identify self-harm records and their date. In the linked HES A&E data, we used a variable indicating the reason for an A&E episode, with value ‘30’ representing deliberate self-harm. In the linked HES APC data and HES operations data, we included episodes with primary or other diagnoses as ICD-10 codes: X6, X7, X80, X81, X82, X83, X84 or Y87.0 as self-harm records, with event date as the hospital episode start date or referral date. In the primary analysis, self-harm records from CPRD and HES were included. In sensitivity analyses, records from CPRD alone were included.

### Exposure period – Quetiapine prescription

The exposure was the period of quetiapine prescription. We identified quetiapine prescriptions from drug issue records in CPRD Aurum and equivalent therapy records in CPRD Gold.

We defined an *exposed* time-period as a window of continuous quetiapine prescribing where consecutive prescriptions were less than 3 months apart. This approach to consecutive prescriptions affords more certainty that a patient continued to be treated, since they had received a repeat prescription. This approach is in line with previous studies using primary care data (Hayes et al., 2019). We defined the length of the quetiapine exposed time-period as the time from the first quetiapine prescription in the period (or follow-up start if exposed at start) to the last quetiapine prescription in the period plus 3 months (or end of follow-up if earlier). To assess the temporal association between self-harm and quetiapine treatment, we divided the exposed time into 0 to <1 month after start of quetiapine prescribing, then the next 1 to <2 months, 2 to <3 months, 3 to <4 months, 4 to <8 months, 8 to <12 months and greater than 12 months exposure.

### Comparison period – Unexposed period without quetiapine prescription

We defined a baseline period without quetiapine prescription as the main comparator. We defined this baseline period as any time up to 12 months before starting quetiapine treatment. We determined the rate of self-harm for the people with an existing personality disorder record during this baseline period and compared it with the exposed periods.

### ‘Pre-exposed’ period – Before quetiapine initiation

**Figure 1.**
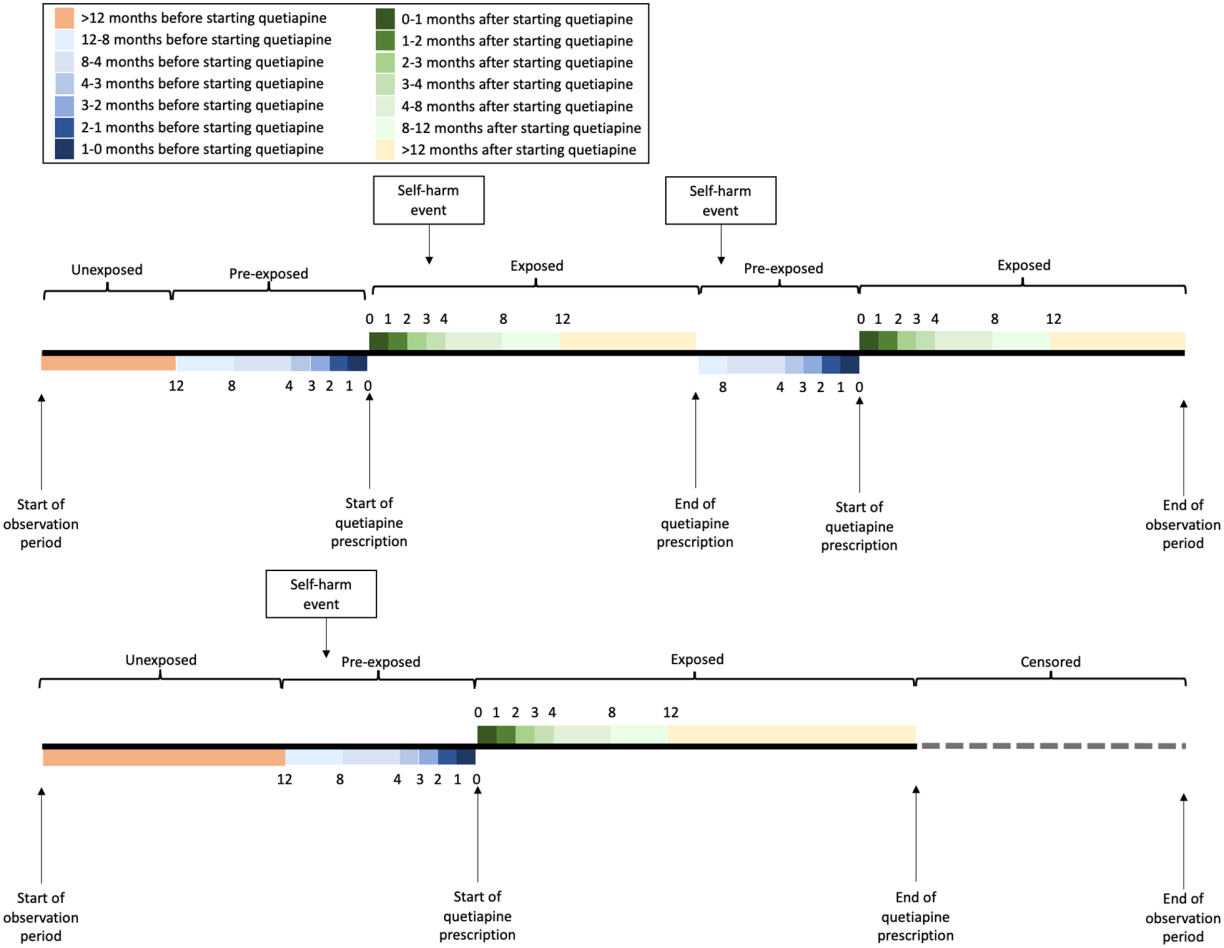
Self-controlled case series design.

We did not include the 12 months before starting quetiapine in this comparator because we wanted to make comparisons with a period of clinical stability. In the SCCS design, outcome events in the period just prior to the exposure are at increased risk of reverse causality and confounding by indication. In this case, it is possible that clinicians prescribe medication such as quetiapine in response to a patient consulting with escalating mental health issues and/or self-harm, and that there is a fluctuating risk of self-harm over this period. Therefore we defined the period 12 months prior to initiating quetiapine as ‘pre-exposed’. We present rates of self-harm in this pre-exposed period in our results, split into the following periods <12 to 8, <8 to 4, <4 to 3, <3 to 2, <2 to1 and <1 to 0 months before commencing quetiapine. Individuals who stopped quetiapine and did not restart during our follow-up period were censored at the end of the final exposed period. Given the complexities of the SCCS design, two example patient treatment trajectories within the SCCS design are shown in Figure 1.

### Time-varying confounders: Age, calendar year and psychotropic prescriptions

We included important time-varying confounders in our fully adjusted analysis: age at the start of the exposure period (categorised as: 0–14, 15–24, 25–34, 35–44, 45–54, 55–64, 65–74, 75+ years), and calendar year at the start of the exposure period (grouped into 2-year bands), exposure to hypnotics, anxiolytics, antidepressants and other antipsychotics (both first and second generation). Exposed and unexposed periods for each of these medication groups were defined using the same approach we applied to quetiapine prescribing. Prescriptions of these medications were identified from drug issue records in CPRD Aurum and equivalent therapy records in CPRD Gold, using the British National Formulary (BNF) as a guide to identify the type of medication: BNF chapter 4.1.1 for hypnotics, BNF chapter 4.1.2 for anxiolytics, BNF chapter 4.3 for antidepressants and BNF chapters 4.2.1; 4.2.2 for other antipsychotics (quetiapine prescriptions excluded). This allowed us to examine the specific effects of quetiapine on self-harm, independent of the effects of any other psychotropic medication.

### Baseline characteristics of people with personality disorders and self-harm

We extracted data including sex, age at study start and area level social deprivation using the Index of Multiple Deprivation which is based on individual’s postcode and grouped into quintiles (Smith et al., 2015). We also extracted data regarding comorbid mental health conditions defined as: SMI, depression, anxiety, phobia, hypochondria, obsessive compulsive disorder, other neurosis, eating disorder, post-traumatic stress disorder. Each of these conditions were identified by the presence of a Read, SNOMED or EMIS code at any time (as described above).

### Statistical analysis

Incidence rates of self-harm were computed for the quetiapine exposed and unexposed periods as the total number of self-harm events occurring in the period divided by the length of the period. We used conditional Poisson regression, with exposure periods nested in individuals and computed incidence rate ratios (IRRs). As described above, the follow-up time was split into exposed periods, pre-exposed periods and an unexposed period (Figure 1).

Initially, we only adjusted for the person’s age at start of each time interval. Then these time intervals were split further according to additional exposure to hypnotics, anxiolytics, antidepressants and other antipsychotics. We included these medication exposures, as well as calendar year at start of the time interval, to enable further adjustment for these time-varying confounders.

### Sensitivity analysis

We repeated the SCCS analysis using self-harm records from CPRD alone (rather than using linked data with episodes of self-harm from secondary care hospitals in HES).The time-window for this analyses was therefore longer (2000–2018) since HES data were only available for a shorter period.

We also completed two supplementary analyses: one that separated out the first unexposed period and subsequent unexposed periods and one that examined the time before, during and after the first quetiapine exposure only.

## Results

The SCCS cohort comprised 1,082 eligible people with a record of personality disorder who were treated with quetiapine during follow-up. They all had at least one episode of self-harm during the study period. There was a total of 1,672 self-harm events in the cohort over a combined total of 2,603.9 person-years of follow-up. The median number of self-harm events per person was 2 (IQR 1–3) and the median follow-up time per person was 2.7 years (IQR 1.3–4.8 years) (Table 1). In the baseline period where people were unexposed to quetiapine, the rate of self-harm was 0.52 events per person year.

**Table 1.** Baseline characteristics of the SCCS cohort.

	*N* = 1082	
	Mean	(*SD*)
Age at study entry* (years)	33.57	(11.11)
	Frequency	(%)
Sex		
Men	279	(25.8)
Women	803	(74.2)
Index of multiple deprivation (patient level)		
1	122	(11.3)
2	163	(15.1)
3	207	(19.1)
4	290	(26.8)
5	298	(27.5)
Missing	2	(0.2)
Personality disorder type		
Paranoid	14	(1.3)
Schizoid	5	(0.5)
Dissocial	31	(2.9)
Emotionally unstable	753	(69.6)
Histrionic	7	(0.6)
Anankastic	15	(1.4)
Anxious	5	(0.5)
Dependent	19	(1.8)
Other	233	(21.5)
Psychiatric comorbidity		
Any SMI*	94	(8.7)
PTSD	105	(9.7)
Eating disorder	175	(16.2)
Depression	978	(90.4)
Anxiety	743	(68.7)
Use of anxiolytics	608	(56.2)
Use of hypnotics	713	(65.9)
Use of antidepressants	929	(85.9)
Use of other antipsychotics	395	(36.5)

SCCS: self-controlled case series; PTSD: post traumatic stress disorder; SMI: severe mental illness.

* Those who receive a SMI code during their follow-up were censored at this date.

**Figure 2.**
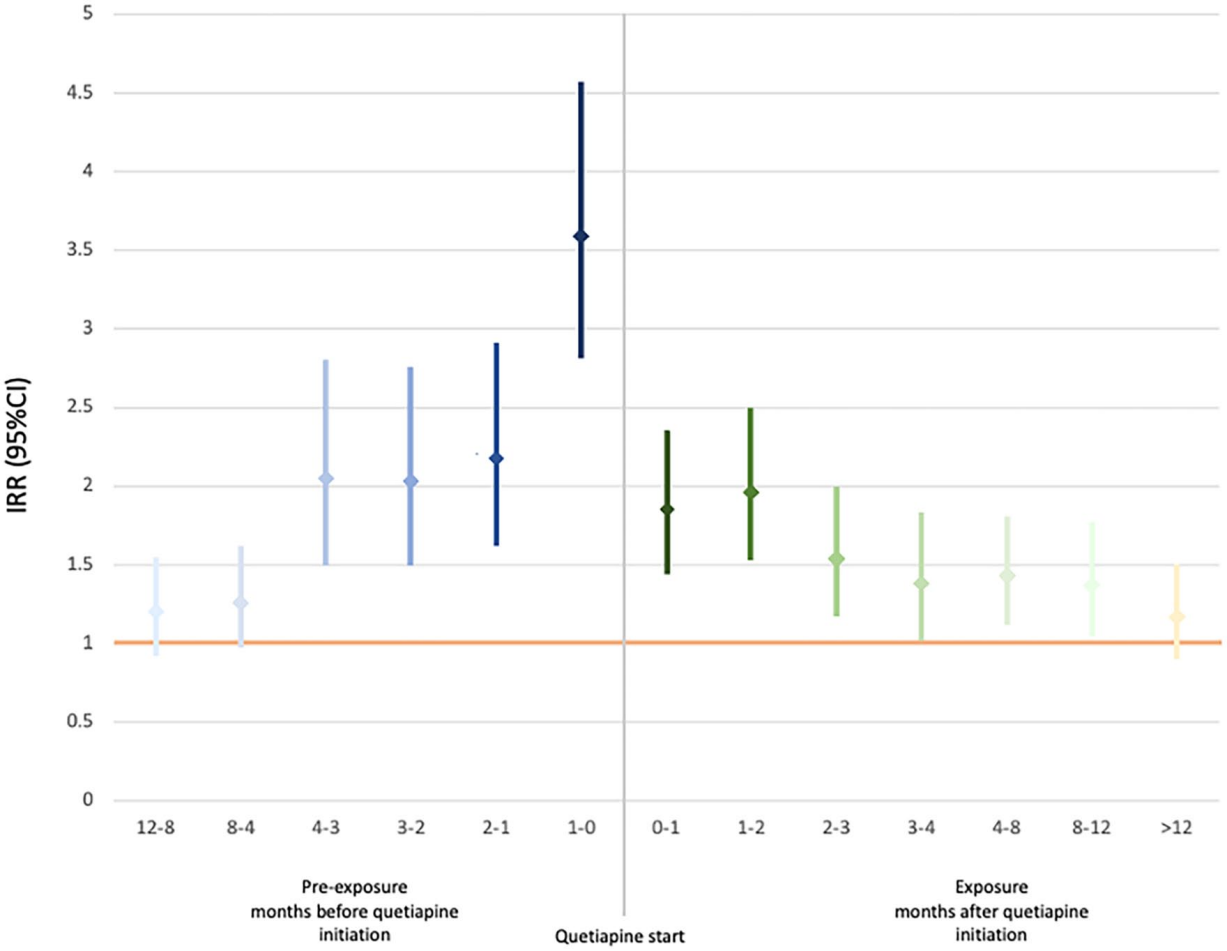
IRRs for self-harm for monthly intervals up to a year before and after start of quetiapine, compared to unexposed period greater than a year before quetiapine initiation. IRR: incidence rate ratio. *All comparisons are with the unexposed period (greater than 12 months before quetiapine initiation, IRR = 1).

Figure 2 shows fully adjusted IRRs and 95% confidence intervals (95% CIs) for self-harm in people prescribed quetiapine relative to the baseline period of 12 months before any quetiapine treatment. Table 2 contains IRRs for self-harm firstly unadjusted, adjusted for age, and then adjusted further for all time-varying confounders including other psychotropic medication.

**Table 2.** Incidence rates and IRRs for self-harm according to exposure to quetiapine.

Exposure		Total self-harm events	Total person-years	Incidence rate per year	Crude IRR	IRR adjusted for age	IRR adjusted for all time-varying confounders*
More than 12 months prior to quetiapine use	Unexposed	344	667.7	0.52	1	1	1
<12–8 months prior to quetiapine use		92	114.6	0.8	1.56 (1.22, 1.97)	1.23 (0.97, 1.57)	1.20 (0.94, 1.53)
<8–4 months prior to quetiapine use		118	141.4	0.03	1.62 (1.30, 2.00)	1.30 (1.04, 1.63)	1.26 (0.99, 1.60)
<4–3 months prior to quetiapine use	Pre-exposed	55	41.1	1.34	2.60 (1.92, 3.46)	2.08 (1.54, 2.80)	2.05 (1.51, 2.79)
<3–2 months prior to quetiapine use		60	46.1	1.3	2.52 (1.89, 3.33)	2.04 (1.53, 2.73)	2.03 (1.51, 2.74)
<2–1 months prior to quetiapine use		71	52.4	1.36	2.63 (2.01, 3.41)	2.15 (1.64, 2.83)	2.18 (1.64, 2.89)
<1–0 month prior to quetiapine use		134	64	2.09	4.06 (3.30, 4.98)	3.45 (2.76, 4.31)	3.59 (2.83, 4.55)
0–<1 month of quetiapine use		163	104.3	1.56	3.03 (2.50, 3.66)	2.07 (1.67, 2.58)	1.85 (1.46, 2.34)
1–<2 months after start of quetiapine use		166	101.9	1.63	3.16 (2.61, 3.82)	2.19 (1.76, 2.72)	1.96 (1.55, 2.48)
2–<3 months after start of quetiapine use		124	99	1.25	2.43 (1.96, 2.99)	1.71 (1.35, 2.16)	1.54 (1.19, 1.98)
3–<4 months after start of quetiapine use	Exposed	89	77.4	1.15	2.23 (1.75, 2.83)	1.52 (1.17, 1.98)	1.38 (1.04, 1.82)
4–<8 months after start of quetiapine use		266	233.4	1.14	2.21 (1.88, 2.60)	1.57 (1.28, 1.92)	1.43 (1.14, 1.79)
8–<12 months after start of quetiapine use		165	162.7	1.01	1.96 (1.62, 2.38)	1.48 (1.18, 1.86)	1.37 (1.06, 1.75)
>12 months after start of quetiapine use		495	697.9	0.71	1.38 (1.20, 1.58)	1.26 (1.03, 1.55)	1.17 (0.92, 1.48)

IRR: incidence rate ratio.

* Age, calendar year, psychotropic medications.

The rate of self-harm was elevated in the year after quetiapine was first prescribed, compared to the unexposed period: IRR at 0–1 months 1.85 (95%CI 1.46–2.34), IRR at 1–2 months 1.96 (95% CI 1.55–2.48) and IRR at 2–3 months 1.54 (95% CI 1.19–1.98). It was only after more than 12 months of quetiapine exposure that the IRR returned to approximately baseline.

In the quetiapine pre-exposure period <4–3 months before quetiapine initiation, the rate of self-harm was more than double the rate in the baseline quetiapine unexposed period (12 months before quetiapine initiation) (fully adjusted IRR 2.03, 95% CI 1.51–2.74). It remained elevated and was highest overall in the month before quetiapine was commenced (fully adjusted IRR 3.59, 95% CI 2.83–4.55).

In our sensitivity analysis using CPRD alone (with no HES linkage), the number of people included increased to more than 2,500. The incidence rates for self-harm in the months before and after the start of quetiapine exposure were reduced compared to the CPRD-HES analysis. However the IRRs for periods when individuals were exposed and pre-exposed to quetiapine were consistent with our primary analysis (Supplemental Table 1). Supplementary analyses suggested a similar increase in self-harm rates in the pre-exposed months before first quetiapine exposure and the months before subsequent quetiapine exposure (Supplemental Table 2). Also, that after the first quetiapine exposure period ended and patients stopped taking quetiapine, self-harm rates returned to baseline 2 months post exposure (Supplemental Table 3).

## Discussion

In a cohort of 1,082 people with a recorded diagnosis of personality disorder, who were prescribed quetiapine and had at least one episode of self-harm recorded in their primary care or HES record, self-harm incidence was elevated in the month after quetiapine initiation, and remained so until greater than a year after the start of treatment. However, self-harm rates peaked in the month before quetiapine initiation (approximately double the IRR for the month after quetiapine initiation), and were elevated up to 4 months before treatment, compared to a baseline period a year or more before first quetiapine prescription. These findings remained after controlling for time-fixed confounders as well as age, calendar period and prescriptions of other psychotropic medications.

There is a clear reduction in rates of self-harm from a month before quetiapine initiation to a month after commencing quetiapine. There is also a continued reduction in the rate of self-harm over the full quetiapine exposure period. This may represent a short-term clinical effect. However, at all times in the first year of quetiapine treatment, self-harm rates remained elevated compared to the baseline period >12 months before quetiapine initiation. It may be unrealistic to expect quetiapine to be associated with a return to baseline rates of self-harm, or to drop below baseline, given the lack of effective pharmacological interventions for self-harm more generally (Hawton et al., 2015).

### Strengths and limitations

We used a SCCS design in linked primary care and hospital records of over 1,000 people. This captured a larger number of self-harm events than had we used primary care records alone. The SCCS design is much less prone to problems of confounding than traditional cohort or case-control designs as comparison is made within, rather than between patients and therefore baseline differences between those included are less important. The method is more efficient than other observational designs and therefore more precise effect estimates can be generated. Our study was based on routine data from UK primary care (CPRD) which has been shown to be broadly representative of the population of the UK. However, included patients had to have at least one self-harm episode presenting to primary or secondary care, and we therefore may have included a more unwell and higher risk population. This is reflected in the high baseline rate of self-harm (0.52 episodes per year). For context, the rate of self-harm in the UK population is around 0.15 episodes per year, according to another CPRD study coving a similar time period (Carr et al., 2016). To be included in the study individuals had to receive a diagnosis of any personality disorder and it is unlikely that clinical coding captures everyone who fulfils the criteria. However, there is likely to be a high degree of sensitivity with respect to the included population because of the additional criteria of quetiapine prescription and self-harm episode. We were unable to examine specific personality disorder subtypes, but in light of changes to ICD-11 we view that as an unnecessary distinction.

Prescription data in CPRD is highly detailed; however, it is likely that some patients were not taking quetiapine during periods we considered them to be exposed. This would result in a reduced effect estimate during the quetiapine exposed period. We may also have misclassified exposed and unexposed periods. We addressed this by splitting up exposure periods by month until 4 months, then in 4-month periods until year. The IRR for self-harm reduced towards no effect over this period. We cannot rule out reverse causation at the time of quetiapine initiation (that is, the episode of self-harm resulted in a quetiapine prescription). An alternative explanation is that additional care may have reduced the immediate risk of self-harm. Information about the receipt of other health care, such as crisis resolution and home treatment team support, is not well captured in primary care or HES records. However, it is unlikely that any such support would continue past the first few months of quetiapine exposure and we observe a continued reduction in rates of self-harm over a prolonged period. Our supplementary analysis that separated out higher risk unexposed periods, when such additional care may be in place, did not substantially alter our findings.

We only examined the outcome of self-harm events in people with personality disorders who were prescribed quetiapine. There may be other reasons why quetiapine may be beneficial to people with personality disorders, such as reducing psychological distress or improving sleep. We were unable to study these outcomes as they are not well captured in primary care records, but they may be very important to patients. That said, a self-harm event is very traumatic for the patient and is a hard marker of psychological distress and any intervention that can reduce self-harm would be highly valuable.

## Conclusion

Our study supports the hypothesis that quetiapine may acutely reduce self-harm in people with diagnosed personality disorders. However, self-harm does not reduce to the baseline rate observed 12 months before commencing treatment. One published trial suggests reduced psychological distress and depression at 8 weeks (Black et al., 2014; Lee et al., 2016); our study suggests this may translate into reduced self-harm events in real life. The potential benefit of quetiapine needs to be balanced with the risk of adverse events (including sedation, metabolic abnormalities and weight gain). Clinicians should be very clear what the potential risks and benefits of treatment may be and communicate this explicitly with the patient. If treatment is initiated for short-term symptom management, it should be reviewed and potentially stopped if there is little sustained benefit.

## Supplemental Material

sj-docx-1-jop-10.1177_02698811221131990 – Supplemental material for Association between quetiapine use and self-harm outcomes among people with recorded personality disorder in UK primary care: A self-controlled case series analysis Supplemental material, sj-docx-1-jop-10.1177_02698811221131990 for Association between quetiapine use and self-harm outcomes among people with recorded personality disorder in UK primary care: A self-controlled case series analysis by Joseph F Hayes, Sarah Hardoon, Jessica Deighton, Essi Viding and David PJ Osborn in Journal of Psychopharmacology
