# Supplementary material for: Association between quetiapine use and self-harm outcomes among people with recorded personality disorder in UK primary care: A self-controlled case series analysis
Source: J Psychopharmacol. 2022 Nov 1;36(11):1218–25. doi: 10.1177/02698811221131990 (PMC9643813; doi:10.1177/02698811221131990)
Supplement: sj-docx-1-jop-10.1177_02698811221131990 – Supplemental material for Association between quetiapine use and self-harm outcomes among people with recorded personality disorder in UK primary care: A self-controlled case series analysis [file sj-docx-1-jop-10.1177_02698811221131990.docx]

**Association between quetiapine use and self-harm outcomes among people with recorded personality disorder in United Kingdom primary care: a self-controlled case series analysis**

Supplementary Table 1. Sensitivity analyses – Incidence rates and incidence rate ratios for self-harm recorded in primary care only, according to exposure to quetiapine

| **Exposure** |  | **Total self-harm events** | **Total person-years** | **Incidence rate per year** | **Crude IRR** | **IRR adjusted for age** | **IRR adjusted for all time-varying confounders*** |
| --- | --- | --- | --- | --- | --- | --- | --- |
| More than 12 months prior to Quetiapine use | Unexposed | 897 | 1790.3 | 0.50 | 1 | 1 | 1 |
| 8-12 months prior to Quetiapine use |  | 187 | 294.7 | 0.63 | 1.27 (1.08, 1.48) | 1.13 (0.96, 1.34) | 1.16 (0.98, 1.38) |
| 4-8 months prior to Quetiapine use |  | 275 | 367.4 | 0.75 | 1.49 (1.30, 1.71) | 1.29 (1.11, 1.49) | 1.32 (1.14, 1.54) |
| 3-4 months prior to Quetiapine use | Pre-exposed | 111 | 109.7 | 1.01 | 2.02 (1.64, 2.46) | 1.68 (1.36, 2.06) | 1.76 (1.42, 2.17) |
| 2-3 months prior to Quetiapine use |  | 100 | 121.5 | 0.82 | 1.64 (1.32, 2.02) | 1.35 (1.09, 1.68) | 1.43 (1.14, 1.78) |
| 1-2 months prior to Quetiapine use |  | 160 | 138.9 | 1.15 | 2.30 (1.93, 2.72) | 1.92 (1.60, 2.30) | 2.04 (1.69, 2.45) |
| Up to 1 month prior to Quetiapine use |  | 245 | 168.2 | 1.46 | 2.91 (2.51, 3.35) | 2.51 (2.14, 2.93) | 2.69 (2.28, 3.17) |
| 1st month of Quetiapine use | Exposed | 349 | 268.6 | 1.30 | 2.59 (2.29, 2.94) | 1.82 (1.58, 2.11) | 1.79 (1.53, 2.09) |
| 1-2 months after start of Quetiapine use |  | 292 | 262.6 | 1.11 | 2.22 (1.94, 2.54) | 1.58 (1.36, 1.84) | 1.55 (1.32, 1.83) |
| 2-3 months after start of Quetiapine use |  | 248 | 255.5 | 0.97 | 1.94 (1.68, 2.23) | 1.40 (1.19, 1.65) | 1.39 (1.17, 1.65) |
| 3-4 months after start of Quetiapine use |  | 209 | 201.8 | 1.04 | 2.07 (1.77, 2.41) | 1.48 (1.24, 1.75) | 1.47 (1.23, 1.76) |
| 4-8 months after start of Quetiapine use |  | 589 | 618.4 | 0.95 | 1.90 (1.71, 2.11) | 1.42 (1.25, 1.63) | 1.43 (1.23, 1.65) |
| 8-12 months after start of Quetiapine use |  | 344 | 434.9 | 0.79 | 1.58 (1.39, 1.79) | 1.27 (1.09, 1.48) | 1.28 (1.08, 1.51) |
| More than 12 months after start of Quetiapine use |  | 1117 | 1891.2 | 0.59 | 1.18 (1.08, 1.29) | 1.15 (1.01, 1.32) | 1.16 (1.00, 1.36) |

*age, calendar year, psychotropic medications

Supplementary Table 2 - Incidence rate ratios for self-harm separating out first unexposed periods and subsequent unexposed periods (after a period of quetiapine exposure)

| Exposure |  | Adjusted* IRR |
| --- | --- | --- |
| More than 12 months prior to Quetiapine use | Unexposed - prior to first recorded quetiapine exposure | 1 |
| 12-8 months prior to Quetiapine use | Pre-exposed - prior to first recorded quetiapine exposure | 1.27 (0.96, 1.67) |
| 8-4 months prior to Quetiapine use |  | 1.32 (1.01, 1.71) |
| 4-3 months prior to Quetiapine use |  | 1.69 (1.15, 2.48) |
| 3-2 months prior to Quetiapine use |  | 1.81 (1.25, 2.61) |
| 2-1 months prior to Quetiapine use |  | 2.13 (1.51, 3) |
| 1-0 month prior to Quetiapine use |  | 3.51 (2.64, 4.68) |
| 0-1 month of Quetiapine use | Exposed | 1.74 (1.34, 2.25) |
| 1-2 months after start of Quetiapine use |  | 1.83 (1.41, 2.38) |
| 2-3 months after start of Quetiapine use |  | 1.44 (1.09, 1.9) |
| 3-4 months after start of Quetiapine use |  | 1.29 (0.95, 1.74) |
| 4-8 months after start of Quetiapine use |  | 1.34 (1.04, 1.72) |
| 8-12 months after start of Quetiapine use |  | 1.27 (0.97, 1.68) |
| More than 12 months after start of Quetiapine use |  | 1.09 (0.83, 1.42) |
| More than 12 months prior to Quetiapine use | Unexposed - after recorded quetiapine exposure | 0.84 (0.58, 1.24) |
| 12-8 months prior to Quetiapine use | Pre-exposed - after recorded quetiapine exposure | 0.79 (0.46, 1.35) |
| 8-4 months prior to Quetiapine use |  | 0.92 (0.59, 1.44) |
| 4-3 months prior to Quetiapine use |  | 2.47 (1.52, 4) |
| 3-2 months prior to Quetiapine use |  | 2.13 (1.33, 3.42) |
| 2-1 months prior to Quetiapine use |  | 1.93 (1.23, 3.03) |
| 1-0 month prior to Quetiapine use |  | 3.21 (2.26, 4.55) |

*age, calendar year, psychotropic medications

Supplementary Table 3 - Incidence rate ratios for self-harm according to first exposure to quetiapine

| Exposure |  | Adjusted* IRR |
| --- | --- | --- |
| More than 12 months prior to Quetiapine use | Unexposed - prior to first recorded quetiapine exposure | 1 |
| 12-8 months prior to Quetiapine use | Pre-exposed - prior to first recorded quetiapine exposure | 1.18 (0.98, 1.43) |
| 8-4 months prior to Quetiapine use |  | 1.18 (0.98, 1.42) |
| 4-3 months prior to Quetiapine use |  | 1.45 (1.1, 1.93) |
| 3-2 months prior to Quetiapine use |  | 1.73 (1.33, 2.25) |
| 2-1 months prior to Quetiapine use |  | 1.85 (1.43, 2.39) |
| 1-0 month prior to Quetiapine use |  | 2.35 (1.86, 2.96) |
| 0-1 month of Quetiapine use | Exposed | 1.71 (1.33, 2.18) |
| 1-2 months after start of Quetiapine use |  | 1.82 (1.43, 2.33) |
| 2-3 months after start of Quetiapine use |  | 1.44 (1.11, 1.88) |
| 3-4 months after start of Quetiapine use |  | 1.39 (1.04, 1.87) |
| 4-8 months after start of Quetiapine use |  | 1.47 (1.18, 1.84) |
| 8-12 months after start of Quetiapine use |  | 1.41 (1.09, 1.83) |
| More than 12 months after start of Quetiapine use |  | 1.32 (1.04, 1.67) |
| 0-1 month after stopping Quetiapine | Post-exposure - after first recorded quetiapine exposure | 1.41 (1.02, 1.94) |
| 1-2 months after stopping Quetiapine |  | 1.28 (0.9, 1.81) |
| 2-3 months after stopping Quetiapine |  | 1.4 (0.98, 2) |
| 3-4 months after stopping Quetiapine |  | 1.2 (0.81, 1.78) |
| 4-8 months after stopping Quetiapine |  | 1.16 (0.89, 1.51) |
| 8-12 months after stopping Quetiapine |  | 0.9 (0.66, 1.23) |
| More than 12 months after stopping Quetiapine |  | 0.81 (0.64, 1.04) |

*age, calendar year, psychotropic medications
